# Analysis of Pain and Effectiveness in Digital Block of the First Toe Using Syringe vs. Carpule: Frost’s H vs. Modified Frost’s H Randomized Clinical Trial

**DOI:** 10.3390/jcm13144185

**Published:** 2024-07-17

**Authors:** Ana Mª Rayo-Pérez, Rafael Rayo-Rosado, Rafael Rayo-Martín, María Reina-Bueno

**Affiliations:** Department of Podiatry, Faculty of Nursing, Physiotherapy and Podiatry University of Seville, 41009 Seville, Spain; rafaelrayo@us.es (R.R.-R.); rafarayo2001@gmail.com (R.R.-M.); mreina1@us.es (M.R.-B.)

**Keywords:** digital block, anesthesia, hallux, Frost’s H, effectiveness, pain

## Abstract

**Background**: Currently, there is no scientific evidence regarding pain in the anesthetic block of the first toe according to the method of application. However, clinical evidence has highlighted the use of the carpule due to the low pain it causes during the administration of the anesthetic. Most studies on anesthesia and pain, especially using the carpule and distraction methods, belong to the field of dentistry. **Objective**: To compare the pain and effectiveness between the anesthetic block of the first toe using a carpule and syringe with Frost’s H technique and the modified Frost’s H technique. **Method**: A total of 564 subjects were selected and divided into four groups. Subjects were subjected to experimental conditions (randomization through the Random Allocation Software program 2.0), and divided into group 1 = 138 subjects, corresponding to the block with syringe and Frost’s H, group 2 = 141 subjects, corresponding to the syringe group and modified Frost’s H, group 3 = 141 subjects, corresponding to the carpule group and modified Frost’s H, and group 4 = 144 subjects, corresponding to the carpule group and Frost’s H. The same researcher generated the random allocation sequence, enrolled the participants, and assigned them to the interventions. Each subject was unaware of the anesthetic procedure assigned by the researcher. Outcome parameters were pain after anesthetic infiltration and its effectiveness. **Results**: The anesthetic block with carpule showed a lower pain score compared to the anesthetic block with syringe (2.8 vs. 5.3; *p* < 0.001). However, when analyzing effectiveness, a higher efficacy rate was obtained in the anesthetic blocks performed using the modified Frost’s H technique (97.5% vs. 88.1%; *p* < 0.001). **Conclusions:** The anesthetic block with carpule and the modified Frost’s H technique is less painful and more effective than the traditional anesthetic block.

## 1. Introduction

According to authors like Tumi et al. and Flanagan et al., the most common causes that can justify excessive pain during the anesthetic procedure include the use of inappropriate technique, incorrect needle selection, patient anatomical variations, or involuntary muscle tension [1,2,3,4].

Strazar et al. reported in 2013 that anesthetic injections tend to be the most painful part of invasive procedures and generate the most anxiety for patients. They attribute this to both the needle puncture, which activates Pacinian corpuscles, Ruffini endings, and mechanoreceptors, and the infiltration of the liquid itself, which causes pain due to chemical irritation of free-ending polymodal nociceptors [5].

Ricardo et al. [3] and Flanagan et al. [2] study the influence of needle gauge on pain perception, although McPherson et al. [6] argue that there are no differences between using larger or smaller gauge needles. These authors also note that pain is primarily generated by the infiltration of local anesthetic into the tissues, emphasizing that infiltration should be performed at a constant speed. They highlight the use of topical anesthetics to reduce pain during injection, as well as the use of carpules as an infiltration method, as it reduces needle vibration and facilitates constant drug administration [2,7].

In the case of nail pathology, the commonly used anesthetic procedure is Frost’s H technique. The first puncture is performed in the dorsolateral toe area. Before removing the needle, once the anesthetic drug has been introduced, medialize about 90 degrees to pass the needle under the extensor tendon of the toe and infiltrate the medial area of the toe. The second puncture is performed in the dorsomedial area. Another common technique is the V-technique, which consists of a central puncture over the tendon of the extensor hallucis longus at the base of the first toe. After the initial puncture, the needle is repositioned medially and then laterally. It should be noted that this anesthetic technique often fails to completely anesthetize the plantar nerves, necessitating reinforcement of the area. This is why the modified Frost’s H technique was developed. This technique involves a first puncture in the dorsolateral toe area. The second puncture is performed in the dorsomedial area. Finally, the anesthetic procedure is completed with a third puncture on the medial plantar area of the base of the toe. In both techniques, 1.2 mL of anesthetic solution is injected into each puncture site [8,9,10,11]. The anesthetic procedures described above are based on the nervous anatomy of the first toe. In this sense, the digital nerves run parallel to the bone segments. At the dorsal level, the hallux is innervated in its medial area by the medial dorsal cutaneous nerve, while the lateral area is innervated by the deep peroneal nerve. At the plantar level, it is innervated by the digital branches of the medial plantar nerve [8,9,10,11].

Traditionally, the syringe is used as the infiltration method, although the use of the carpule is also indicated. The carpule is a reloadable metal syringe with specific vials, widely used in the fields of dentistry and dermatology. It allows for precise administration of the drug at a constant speed, thereby reducing pain during injection [8,9,10,11].

Although ultrasound-guided anesthetic blocks have demonstrated greater precision and effectiveness, as well as less pain during the procedure [12], there are currently no references that employ the use of this type of block at the level of the hallux. This is because currently, the equipment does not allow a clear visualization of the nerve structures at this level.

Currently, there is no scientific evidence regarding pain and effectiveness in the anesthetic block of the first toe. However, clinical evidence, especially in the fields of dentistry and dermatology, has highlighted the value of the carpule due to its ability to reduce patients’ perception of pain [1,8,13].

The aim of this study is to compare pain and effectiveness in digital anesthesia of the hallux using different infiltration methods, such as the syringe and carpule, as well as two techniques, Frost’s H and modified Frost’s H. Secondary objectives include comparing pain differences based on the subjects’ sex and age.

## 2. Materials and Methods

### 2.1. Design and Sample

A randomized, single-blind clinical trial was conducted in accordance with the Consolidated Standards of Reporting Trials (CONSORT) guidelines [14]. 

The clinical trial was registered with Clinical Trials under reference NCT06352255. The study was also approved by the Bioethical Research Committee of the University of Málaga (207-2023-H) and authorized by the Clinical Area Management of Podiatry at the University of Seville. 

The study sample consisted of patients treated at the authorized clinical center Clínica Rayo in Seville and was conducted between September 2021 and December 2023. Participants provided written consent. A total of 564 subjects were selected and divided into four groups. Patients were subjected to experimental conditions (randomization through the Random Allocation Software program 2.0), and divided into group 1 = 138 subjects, corresponding to the syringe and Frost’s H technique; group 2 = 141 subjects, corresponding to the syringe and modified Frost’s H technique; group 3 = 141 subjects, corresponding to the carpule and modified Frost’s H technique; and group 4 = 144 subjects, corresponding to the carpule and Frost’s H technique. 

The same researcher generated the random allocation sequence, enrolled the participants, and assigned them to the interventions. Each subject was unaware of the anesthetic procedure assigned by the researcher. For this purpose, a sheet of numbers from 1 to 100 was used, which would correspond to a group. Before starting, the patient was asked for a random number to randomize him into one of the four groups. During the anesthetic procedure, a visual censure was placed between the patient and the researcher. During the investigation all the participants were blinded, since they did not know what types of procedure were assigned. The only information that they were given was that the research would evaluate the effectiveness of four anesthetic procedures.

Inclusion criteria were ages 18 to 70 years and any sex. Exclusion criteria included being pregnant or possibly pregnant, having hypersensitivity to local anesthetics, and having severe systemic disease or neurological disorders that would prevent proper participation in the study. Outcome parameters were pain after anesthetic infiltration and its effectiveness.

### 2.2. Procedure

To minimize biases secondary to the procedure, all anesthetic procedures were performed by the same investigator, a foot surgery specialist with over 10 years of experience in the field of digital anesthesia, following the protocol described below. Previously, the anesthetic procedures have been repeatedly tested under supervision. 

Before the anesthetic technique, the toe was cleaned with 2% chlorhexidine for aseptic preparation. The procedure was always performed by the same professional with more than 10 years of experience. A sterile 27-gauge (G) needle, measuring 40 × 25 mm (mm), was used to infiltrate 3.6 milliliters (mL) of 2% mepivacaine, divided equally among each infiltration site. For Frost’s H technique, a wheal was created on the medial, dorsal, and lateral sides of the toe, while for the modified Frost’s H technique, a wheal was created on the medial, lateral, and plantar sides of the toe.

**Figure 1:**
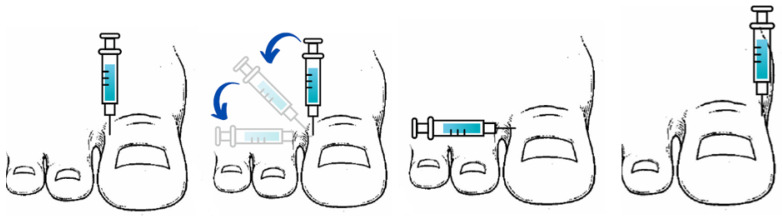
Anesthetic block using Frost’s H technique. Source: Own work.

To perform the Frost’s H anesthetic technique, the first puncture is performed in the dorsolateral toe area. Before removing the needle, once the anesthetic drug has been introduced, medialize about 90 degrees to pass the needle under the extensor tendon of the toe and infiltrate the medial area of the toe. The second puncture is performed in the dorsomedial area. It should be noted that these injection points were made at a 90° angle relative to the axis of the toe to ensure proper anesthetic block of the deep peroneal nerve and the digital plantar nerve (Figure 1).

**Figure 2:**
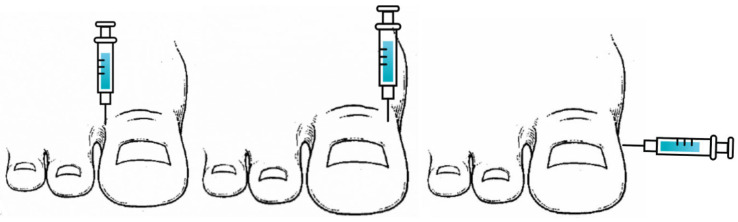
Anesthetic block using the modified Frost’s H technique. Source: Own data.

In the case of the modified Frost’s H anesthetic technique, a first puncture is performed in the dorsolateral toe area. The second puncture is performed in the dorsomedial area. Finally, the anesthetic procedure is completed with a third puncture on the medial plantar area of the base of the toe. As with Frost’s H technique, the punctures are performed at a 90° angle to the longitudinal axis of the toe (Figure 2).

### 2.3. Outcome Measurements

Another investigator was responsible for data collection and outcome measurement.

Pain was recorded immediately after the entire anesthetic process, providing the patient with a visual analog scale (VAS). Using a numerical scale, the patient rated the pain from 0 to 10, with 10 being the worst pain endured [15]. To control the effectiveness of the technique, a puncture was performed in the center of the hallux pulp with a 23G needle measuring 25 × 0.6 mm, five minutes after the injection [16].

### 2.4. Sample Size Calculation

For the statistical analysis, the sample size was calculated.

A proportion (p) equal to 0.5 was selected to establish the maximum sample size estimation. A confidence level of 95% and a confidence interval precision (d) of ±6% were established.

The result of the sample size calculation corresponded to a minimum of 267 cases per group. These groups were further divided into two subgroups similar in terms of age and sex, with a minimum of 141 subjects per case.

### 2.5. Statistical Analysis

Qualitative variables were expressed through their frequencies and percentages; quantitative variables were expressed in terms of mean and standard deviations, and the 95% confidence interval (CI; upper and lower limits) for parametric data, as well as the median and interquartile range (IQR) for non-parametric data. The Mann–Whitney U test for independent samples was applied for variables related to pain and efficacy.

Statistical analysis was performed using the statistical software IBM SPSS 22.0 (IBM; Armonk, NY, USA). Statistically significant differences were established at *p* < 0.05 with a 95% CI.

## 3. Results

### 3.1. Descriptive Data

The sample consisted of a total of 564 subjects: 411 women (72.9%) and 153 men (34.1%). Regarding laterality, 289 were right feet (51.24%) and 275 were left feet (48.76%). The groups were similar in terms of age, gender, and laterality. The average age is shown in Table 1:

The characteristics of the study subjects for each group are presented in Table 2.

### 3.2. Outcome Measurements

Pain and effectiveness were the main indicators described previously. For the group receiving the anesthetic block with syringe and Frost’s H technique, the pain score was 5.3 points, while the group with the anesthetic block using a carpule reported a pain score of 2.8 points. When subdividing the groups according to the anesthetic technique employed, the scores were 5.3 points for the syringe and Frost’s H group, 5.4 points for the syringe and modified Frost’s H group, and 2.8 points for both the carpule Frost’s H and modified Frost’s H groups. The difference in pain between the four groups was statistically significant (Kruskal–Wallis test for independent samples, *p* < 0.001).

In terms of efficacy among the four groups, significant differences were found (*p* < 0.001), with the group using the modified Frost’s H technique with carpule achieving the highest effectiveness in the anesthetic block.

Regarding pain comparison across different groups, the results are shown in Table 3.

For comparisons with significant differences, the effect sizes found are all moderate to large (r^1^ > 0.5), suggesting that the observed differences are substantial and have practical relevance. Values of *p* < 0.001 in several comparisons indicate a low probability of making Type I errors, and high effect sizes suggest a low probability of making Type II errors. In comparisons where *p* > 0.999, although there is no risk of Type I error, the risk of Type II error is higher, implying that a real difference might not have been detected.

The results for secondary indicators such as age did not show statistically significant differences between the groups (*p* = 0.762). Similarly, no differences were found regarding laterality (*p* = 0.197) and sex (*p* = 0.054).

## 4. Discussion

In our study, we analyzed 564 subjects, predominantly women, with the aim of assessing the pain and effectiveness of different anesthetic block techniques for the hallux. It was found that the block using a syringe produced more pain than the block using a carpule, and the modified Frost’s H technique was more effective than the classic Frost’s H technique (*p* < 0.001). However, no significant differences were observed in age between the groups.

Regarding the anesthetic technique, Sánchez-Hernández et al. reported 25 cases where a V-block anesthetic was applied as an alternative to the Frost’s H technique. They reported no adverse effects in any of the cases. In terms of effectiveness, they found that in 21 out of the 25 cases, the anesthetic block was effective. As a limitation of their study, they mentioned that there were no previous studies comparing the effectiveness of this technique with others, so it cannot be demonstrated that the V-technique is superior to others [8].

The same author analyzed the efficacy of the V-technique compared to the Frost’s H technique, obtaining 87.5% effectiveness versus 67%. They concluded that the V-technique is more effective than the Frost’s H technique. However, it should be noted that the study only included 16 subjects, which limits the ability to determine effectiveness accurately [16].

Regarding pain, Browne et al. [17] in their 2000 study analyzed the use of prilocaine anesthetic cream and the application of anesthesia at a 90° angle to the toe. They analyzed a total of 55 patients with onychocryptosis, divided into two groups. In both cases, they applied a total of 6 mL of 0.5% bupivacaine using a 25G needle. They administered 3 mL of local anesthetic to each side, applying an initial bolus to the plantar area and progressively administering the anesthetic to the dorsal area of the toe. Pain was assessed using a visual analog scale (VAS). The results showed no differences in age or sex of the subjects, while our study obtained higher pain scores in young adults. Statistically significant results were obtained for pain (*p* < 0.001) [17].

Hayward et al. [18] analyzed 20 patients undergoing first toe anesthetic block with 2% lidocaine in a 2006 study. Prior to anesthetic infiltration, cryoanalgesia was applied to reduce the pain of the injection. The results showed that the application of local cold significantly reduced injection pain (*p* < 0.001). The average VAS scores were 5.7 without any prior treatment and 1.6 with ice application. In our case, without any prior treatment, we obtained a pain score of 2.8 for the carpule anesthetic application and 5.3 for the syringe anesthetic application. Therefore, similar results were obtained for anesthetizing without any prior treatment and performing the block with a syringe [18].

Ricardo et al. [3] in their 2021 study analyzed various aspects that may influence greater pain perception during the anesthetic procedure in nail surgery. They mentioned methods such as buffering the anesthetic solution, infiltrating slowly, inserting the needle at a 90º angle to the skin, using devices like the carpule to reduce needle vibration, or applying local cold. The results indicated less pain sensation in patients who received cold air using a constant device that applied air at −30 °C and a constant flow of 500 to 1000 L/minute [3].

When analyzing the infiltration method, it is necessary to extrapolate the results to dentistry and dermatology, as there are no studies comparing existing digital block techniques in the foot. Kuscu et al. [19] associated pain during the anesthetic procedure with patient anxiety and/or the injection device. They used two types of devices, the traditional plastic syringe and the carpule. They analyzed a total of 41 subjects, divided into a single study, assigning the anesthetic method sequentially. The results indicated that anxiety played a significant role in pain perception, even more than the device used for local anesthetic administration. They proposed promoting anxiety control through behavioral management techniques.

Other authors, such as Mittal et al. [20] in 2015, reported on pain in local anesthesia using a plastic syringe and carpule. They analyzed a total of 100 subjects, divided into groups equal in terms of age and sex. According to the VAS, patients who experienced less pain were those assigned to the carpule group (*p* < 0.005). When comparing sexes, women perceived slightly more pain than men. However, in our study, women reported lower VAS scores [20].

Libonati et al. in 2018 [15] conducted a systematic review on the different anesthetic techniques in dentistry and their relationship with anxiety and pain experienced by patients. Patients associated the injection with pain due to the trauma caused by the needle; this pain was also associated with the sudden distension of the tissues by the anesthetic agent. They concluded that this computerized system produces significantly less pain perception compared to traditional methods, although further research is needed.

More recently, in 2019, Oliveira et al. [13] stated that pain during local anesthetic administration is the main reason for patient fear and anxiety. They analyzed different methods to minimize this pain and determine the pain level during injection with different methods. They analyzed a total of 41 subjects, with one group receiving anesthesia with a syringe and another group with a carpule. In both cases, a short 30G needle was used. Pain was assessed using a VAS. The results indicated no statistically significant differences between the two anesthetic methods (*p* > 0.005), while the final duration of the anesthetic block was longer with the syringe application (*p* < 0.001). In our case, given the scarcity of articles comparing both infiltration methods, we analyzed a total of 564 patients undergoing digital anesthesia. In both groups, syringe and carpule, the drug was applied under the same environmental conditions, the same needle incidence angle, and the same gauge (27G), as well as similar infiltration pressure. Our results showed statistically significant differences between the two groups in terms of pain (*p* < 0.001) and effectiveness (*p* = 0.006).

The authors Min et al. [21] in 2024 provided a comprehensive overview of the literature on new dental anesthetics and associated devices designed to relieve pain during dental procedures. They suggested that computer-controlled local anesthetic delivery systems reduce injection pain and discomfort. However, evidence for other devices was limited; development and research into innovative technologies to reduce dental pain and anxiety provide opportunities for improved patient care in dental practice. They concluded that efficient pain management is essential for technique effectiveness, treatment success, and patient adherence.

Vitale et al. [22] in 2023 compared the discomfort felt by pediatric patients during dental local anesthesia administered using either a carpule or the computerized device. According to this study, the computerized anesthesia device is effective in reducing pain during anesthetic injection in children, making it a promising tool for pediatric dental procedures.

Arendt et al. [23] used 23G, 27G, 30G, and 32G gauges to perform injections on a total of 120 patients divided into four equal groups. In all cases the same type of local anesthetic was administered, with the same environmental conditions and with the same device. As a result, statistically significant differences were obtained between the four groups, with those anesthetic blocks performed with larger gauge needles being less painful (*p* < 0.001).

Among the limitations of this study, we found that there is little literature on the use of the carpule as a tool for the anesthetic block in the foot. Additionally, there are few references on pain after injection. However, in our daily practice, the carpule is becoming increasingly popular, especially for the digital block, due to its ergonomic design and the ease of anesthesia application, making it an almost painless method.

Application of the results of this research could minimize pain and improve the effectiveness of first toe anesthetic procedures.

## 5. Conclusions

The anesthetic block using a carpule and the modified Frost’s H technique is less painful and more effective than the anesthetic block using a syringe.

There were no significant differences in pain during the anesthetic procedure according to the age and sex of the subjects.

## Figures and Tables

**Table 1:** Age characteristics of the study sample by group distribution. IC Confidence interval. Source: Own data.

	GROUP 1 N = 138 (24.5%)			GROUP 2 N = 141 (25.0%)			GROUP 3 N = 144 (25.5%)			GROUP 4 N = 141 (25.0%)			*p*
	N	%	IC95%	N	%	IC95%	N	%	IC95%	N	%	IC95%	
**Sex**													0.054
**Women**	93	67.4	59.6–75.2	109	77.3	70.4–84.2	98	68.1	60.4–75.7	111	78.7	72.0–85.5	
**Men**	45	32.6	24.8–40.4	32	22.7	15.8–29.6	46	31.9	24.3–39.6	30	21.3	14.5–28.0	
**Laterality**													0.197
Right foot	66	47.8	39.5–56.2	80	56.7	48.6–64.9	66	45.8	37.7–54.0	77	54.6	46.4–62.8	
Left foot	72	52.2	43.8–60.5	61	43.3	35.1–51.4	78	54.2	46.0–62.3	64	45.4	37.2–53.6	

**Table 2:** Characteristics of the study sample by group distribution. Mean M; Standard Deviation SD. Source: Own data.

	GROUP 1			GROUP 2			GROUP 3			GROUP 4			*p*
	M	SD	IC95%	M	SD	IC95%	M	SD	IC95%	M	SD	IC95%	
**AGE**	40.2	19.2	37.0–43.4	41.9	19.8	38.6–45.2	39.7	19.6	36.4–42.9	40.1	18.6	37.0–43.2	0.762
**PAIN**	5.3	1.8	4.9–5.6	5.4	1.2	5.2–5.6	2.8	1.4	2.6–3.1	2.8	1.3	2.6–3.0	<0.001
**EFECTIVENESS**	79.6%		72.8–86.3%	96.4%		93.4–99.5%	90.3%		85.4–95.1%	98.6%		96.6–100%	<0.001

**Table 3:** Comparisons of outcome measurements between both groups. Source: Own data.

	Syringe + Frost’s H Group	Syringe + Modified Frost’s H Group	Carpule + Frost’s H Group	Carpule + Modified Frost’s H Group
**Syringe + Frost’s H Group**	-	*p* > 0.999	*p* < 0.001 r^1^ = 0.621	*p* < 0.001 r^1^ = 0.634
**Syringe + Modified Frost’s H Group**	*p* > 0.999	-	*p* < 0.001 r^1^ = 0.700	*p* < 0.001 r^1^ = 0.712
**Carpule + Frost’s H Group**	*p* < 0.001 r^1^ = 0.621	*p* < 0.001 r^1^ = 0.700	-	*p* > 0.999
**Carpule + Modified Frost’s H Group**	*p* < 0.001 r^1^ = 0.634	*p* < 0.001 r^1^ = 0.712	*p* > 0.999	-

r^1^ de Rosenthal.

## Data Availability

The original contributions presented in the study are included in the article, further inquiries can be directed to the corresponding author.
